# Editorial: Women in environmental physiology 2022

**DOI:** 10.3389/fphys.2023.1175366

**Published:** 2023-03-08

**Authors:** Ingrid Eftedal, Simona Mrakic-Sposta, Morin Lang

**Affiliations:** ^1^ Norwegian University of Science and Technology, Trondheim, Norway; ^2^ Institute of Clinical Physiology, National Research Council (IFC-CNR), Milano, Italy; ^3^ Department of Rehabilitation Sciences and Human Movement, Faculty of Health Sciences, Universidad de Antofagasta, Antofagasta, Chile

**Keywords:** extreme environments, high altitude, human performance, hypoxia, microgravity, sexual dimophism, women

The United Nations has declared gender equality and science as essential for humanity to reach its goals for sustainable development. Still, women account for only 1/3 of the worlds’ researchers and 1/8 of the members of national science academies (Unesco, 2021). Also, longstanding underrepresentation of female populations as study participants in biology, physiology and medicine in extreme environmental conditions limits our knowledge of mechanisms and treatments important for women’s’ health (Nowogrodzki, 2017). The Women in Environmental Physiology Research Topic contains a collection of scientific papers that highlight work led by women, to narrow the gender gap and invite us to break archaic paradigms that indicate that it is more difficult to study women. The papers address responses and performance at high altitude, in aeronautics, space, and Mars-like conditions, including physical and cognitive function and stress in hypobaric hypoxia, microgravity and in isolated and confined environments. Most of the papers present studies of women’s responses to challenging environments.

In a study of women’s mood at high altitude, Alcantara-Zapata et al. examined a possible role of serotonin-dependent signal transduction *via* the tryptophan-melatonin axis in responses to hypobaric hypoxia. Given that women have lower peripheral serotonin and higher prevalence of anxiety and depression at sea level than men and that altitude exacerbates the risk of mood disorders, the authors discussed sexual dimorphism of serotonin availability in mood regulation at high altitude. They argued that the tradeoff between metabolic demands of thermoregulation and neural circuitry repair, both of which involve the tryptophan-melatonin axis, may leave women at higher risk than men. Their findings support monitoring of women’s hormone cycles and suggest a role for trials of selective serotonin reuptake inhibitors to protect their health, wellbeing, and safety of women sojourning at high altitude. These points should be considered especially now that job offers for women in at high altitude mining are increasing.

Furter on the theme of sex-specific responses to hypoxia, Páez et al. studied lactate production and pulmonary ventilation during anaerobic sprint tests at sea level and high altitude, comparing the outcomes for young women and men. They found that while lactate levels did not significantly differ between women and men for tests performed at sea level, women experienced a larger increase in lactate in the first 24 h at high altitude. Women’s ventilatory response decreased more than that of men, causing higher work of breathing. They results encourage further investigation into sexual dimorphism of fatigue-induced metaboreflex of the respiratory muscles and aerobic–anaerobic transition, which would be important when planning training and performance at high altitude.

Moving on to aerospace medicine, Vento et al. address the scarcity of research with female participants within the tactical aviation community. In a retrospective study comprising data from six studies of acute hypoxic exposure, they explored sex difference in physiological and cognitive performance. They found that while sex, age, and body mass index were not robust predictors of responses to the hypoxic challenge, being a woman was associated with lower peripheral oxygen saturation and higher likelihood of headaches, again indicating sex-dependent dimorphisms in responses to hypoxia albeit with high intra-individual variability.

Finally, the topic contains two papers that explore the physiology and psychology in simulated microgravity and Mars-like conditions. Both have female lead authors. Diaz-Artiles et al. present a study in which they explore young adults’ ability to adapt to altered gravity during a complex bimanual force coordination task, and possible constraints that could influence their coordination. The study group consisted of an equal number of female and male participants. Results indicated that subjects were able to transfer their training performance during the Earth condition to the microgravity condition without additional training. The conclusion of the study was that multi-frequency coordination was effectively performed in microgravity.

In a comprehensive study of stress during 8–12 months in Mars-like conditions, Rosenberg et al. examined the processes and phases involved in adaptation to life and work in isolated and confined environments. Through a combination of metabolite profiling of steroids, stress hormones, neurotransmitters, antioxidative damage, along with wearable monitoring, and self-reported ratings of stress, mood, social participation, and perceived health, the participants’ responses agreed with theories previously described; with elevated alertness during the initial phase, adverse conditions developing after the halfway point, and finally a period of volatility that lasted until the mission ended.

Taken together, the data presented in this Research Topic may benefit both women and men who venture into environments that challenge their capacity for adaptation at multiple levels: biobehavioral, cognitive, psychosocial, molecular, and physical performance. This may further inform targeted prevention and treatment strategies, increasing safety for all through more comprehensive understanding of human physiology. Since women are increasingly a growing force in many extreme environments, from flight missions, to space, to hypoxic or hyperoxic environments, an ethically and sensitive approach to the participation of women in research should be a commitment of policy stake holders and the entire scientific research community. Finally, an integrative and multidisciplinary approach should be fostered in future simulated and in-field studies.
